# Parthenolide Restores Testosterone Biosynthesis After Nanoplastic Exposure by Blocking ROS-Driven NF-κB Nuclear Translocation

**DOI:** 10.3390/antiox14111315

**Published:** 2025-10-31

**Authors:** Peng Zhao, Hao Yan, Runchang Wang, Jie Zhao, Xiangqin Zheng, Dinggang Li, Xitong Guo, Fengming Ji, Chunlan Long, Lianju Shen, Guanghui Wei, Shengde Wu

**Affiliations:** 1 Department of Urology, Children’s Hospital of Chongqing Medical University, National Clinical Research Center for Child Health and Disorders, Ministry of Education Key Laboratory of Child Development and Disorders, Chongqing 400014, China; 2023130287@stu.cqmu.edu.cn (P.Z.); 2023111108@stu.cqmu.edu.cn (H.Y.); 2023130286@stu.cqmu.edu.cn (R.W.); 2022130064@stu.cqmu.edu.cn (J.Z.); 2023140329@stu.cqmu.edu.cn (X.Z.); 2024130292@stu.cqmu.edu.cn (D.L.); 2024130291@stu.cqmu.edu.cn (X.G.); 2024440236@stu.cqmu.edu.cn (F.J.); 481113@cqmu.edu.cn (C.L.); 482061@hospital.cqmu.cn (L.S.); u806806@cqmu.edu.cn (G.W.); 2 Children Urogenital Development and Tissue Engineering of Chongqing Education Commission of China, Chongqing 400014, China

**Keywords:** polystyrene nanoplastics, oxidative stress, steroidogenesis, parthenolide, testosterone

## Abstract

Nanoplastics are pervasive contaminants that adversely affect male reproductive function, yet the molecular basis of polystyrene nanoplastic (PS-NP) toxicity in immature testes and effective preventive strategies remain unclear. Here, male mice (postnatal days 22–35, PND 22–35) and TM3 Leydig cells were exposed to graded PS-NPs, followed by transcriptomic profiling to identify differentially expressed genes (DEGs). Candidate therapeutics were prioritized using Connectivity Map (CMap) analysis and molecular docking, and protein interactions were examined by co-immunoprecipitation (Co-IP). PS-NPs accumulated in immature testes, eliciting excessive reactive oxygen species (ROS) and activation of NF-κB. These events coincided with the downregulation of steroidogenic enzymes (CYP11A1 and StAR) and disruption of testicular microarchitecture. In TM3 cells, PS-NPs suppressed testosterone synthesis in a concentration-dependent manner; this effect was fully reversed by pretreatment with N-acetylcysteine (NAC) or Bay 11-7082. Co-IP demonstrated p65–steroidogenic factor-1 (SF-1) binding consistent with formation of a transcriptional repressor complex targeting steroidogenic genes. CMap and docking analyses nominated parthenolide (PTL) as a candidate inhibitor of NF-κB nuclear translocation (predicted binding affinity, −6.585 kcal/mol), and PTL mitigated PS-NP-induced impairment of testosterone synthesis in vitro. Collectively, these data indicate that PS-NPs disrupt testosterone biosynthesis in immature testes through the ROS/NF-κB/p65–SF-1 axis, while PTL emerges as a candidate small molecule to counter nanoplastic-associated reproductive toxicity. These findings underscore translational relevance and support future evaluation under chronic low-dose exposure conditions, including in vivo validation of PTL efficacy, pharmacokinetics, and safety.

## 1. Introduction

The rising global incidence of male infertility has become a major public health concern, attracting substantial scholarly interest in recent years [1]. Research on male reproductive health has intensified, especially in regions with limited healthcare resources, where infertility rates are disproportionately high [2]. Accumulating evidence indicates that environmental exposures are key risk determinants of male reproductive dysfunction. These exposures encompass diverse stressors, including chemical pollutants, climate change, and plastic contamination [3]. Among these, plastic pollution has emerged as a critical and escalating threat. Over the past several decades, plastic production and utilization have expanded markedly, becoming indispensable to modern economies. A comprehensive global assessment estimated that by 2015, approximately 8.3 billion metric tons of virgin plastic had been manufactured worldwide, generating 6.3 billion metric tons of waste. Alarmingly, only 9% of this waste was recycled, 12% was incinerated, and the remaining 79% accumulated in landfills or dispersed into natural environments. If current production and waste-management trajectories persist, projections suggest that up to an estimated 12 billion metric tons of plastic waste will accumulate in landfills or natural ecosystems by 2050 [4].

Plastics can migrate from food contact materials (FCMs)—including packaging—into foods, thereby contributing to human exposure. In environmental settings, plastic items progressively fragment into microplastics (MPs) and nanoplastics (NPs), which can enter the human body through multiple routes. Dietary intake is a primary exposure route because particles may be ingested via contaminated drinking water and the food chain [5]. Polystyrene (PS) is widely used across industrial and consumer applications and has therefore attracted substantial toxicological scrutiny in biomedical and environmental sciences. Polystyrene nanoparticles (PS-NPs) have been reported to affect reproductive systems through multiple mechanisms, with pronounced effects on testicular function. Exposure to NPs can trigger excessive generation of reactive oxygen species (ROS), producing oxidative stress that damages germ cells and thereby impairs reproductive capacity [6]. Studies in freshwater organisms such as *Daphnia pulex* suggest that NPs hinder growth and reproduction by perturbing mitogen-activated protein kinase (MAPK)–hypoxia-inducible factor-1 (HIF-1)/nuclear factor κB (NF-κB)-mediated antioxidant defenses [6]. In parallel, the biodistribution and accumulation of NPs within organisms can directly compromise reproductive systems. In zebrafish, PS-NPs accumulate in the gonads, intestines, liver, and brain, where they elicit oxidative stress and tissue injury. Such accumulation has been associated with impaired reproductive function, behavioral dysregulation, and reduced survival fitness [7]. Consistent findings in mice show testicular accumulation of PS-NPs, accompanied by cellular apoptosis and inflammatory responses that collectively threaten reproductive health [8].

The NF-κB signaling pathway orchestrates a broad array of biological processes, particularly innate and adaptive immune responses [9]. Emerging evidence indicates that NPs can activate NF-κB, thereby precipitating tissue injury across multiple organs. In murine models, exposure to PS-NPs upregulates pro-inflammatory mediators and initiates acute inflammatory responses [10]. Complementary data from zebrafish demonstrate that PS-NPs modulate inflammatory programs through ROS-driven NF-κB activation. In these models, PS-NP exposure elevates oxidative stress and increases the expression of pro-inflammatory cytokines [11]. Within the reproductive system, PS-NP exposure has been linked to compromised testicular function via oxidative stress and inflammation [12]. However, the specific molecular nodes by which NPs suppress testosterone biosynthesis in immature testes remain insufficiently defined.

PTL is a natural sesquiterpene lactone predominantly isolated from feverfew (*Tanacetum parthenium*) [13]. Historically, this medicinal herb has been used to manage inflammatory and pain-related conditions. PTL has the molecular formula C_15_H_18_O_3_ and a molecular weight of 246.3 g/mol [14]; it is a white to pale-yellow crystalline solid that dissolves readily in common organic solvents [15]. Extensive pharmacological studies indicate that PTL exerts anti-inflammatory, antitumor, and immunomodulatory activities. PTL is a well-characterized inhibitor of the NF-κB signaling pathway and displays antioxidant activity that limits ROS-driven cellular injury [16,17]. These properties provide a mechanistic rationale for using PTL to interrogate or mitigate ROS/NF-κB-dependent processes in the testis.

Here, we established in vivo and in vitro models of PS-NP exposure to interrogate the mechanisms underlying immature testicular injury. We focused on the ROS–NF-κB axis as a putative driver of steroidogenic dysfunction and evaluated its impact on testosterone biosynthesis. We further tested PTL, a sesquiterpene lactone, as a targeted intervention to inhibit ROS-driven NF-κB nuclear translocation and to rescue PS-NP-induced defects in testicular function.

## 2. Materials and Methods

### 2.1. PS-NPs Source

A stock suspension of 60 nm (1% *w*/*v*), green-fluorescently labeled polystyrene nanoparticles (PS-NPs) was purchased from Base Line Chromtech Research (Tianjin, China). At 25 °C, the hydrodynamic diameter, polydispersity index (PDI), and zeta potential (ζ-potential) of particles dispersed in distilled water were determined by laser Doppler velocimetry (LDV) using a Zetasizer Pro Blue particle size analyzer (Malvern Instruments, Malvern, UK) after brief ultrasonication. The PDI describes the breadth of the size distribution: values < 0.05 denote highly monodisperse suspensions with very narrow distributions; values between 0.05 and 0.70 indicate acceptable, relatively uniform dispersions with moderate polydispersity; and values > 0.70 reflect very broad distributions for which the Z-average loses interpretive value and the data reliability is reduced [18]. As a general guideline for colloidal stability, ζ-potential values between ±10 mV indicate that nanoparticles are electrically neutral or near neutral. This results in weak electrostatic repulsion and a tendency to aggregate or settle, which suggests instability. In contrast, ζ-potential values exceeding ±30 mV show that nanoparticles possess a sufficiently strong surface charge, generating enough electrostatic repulsion to overcome van der Waals attraction and thereby indicating good stability [19,20,21]. The working solution of PS-NPs was prepared by adding 20 µL of the original stock solution (1% *w*/*v*) to 1980 µL of deionized water (ddH_2_O) and mixing thoroughly. An aliquot of 10 µL was deposited onto a CaF_2_ window; after air-drying, the polystyrene structure was analyzed using 532 nm Raman spectroscopy (LabRAM Odyssey; HORIBA, Kyoto, Japan). Another 10 µL was placed at the center of a silicon wafer, allowed to dry naturally, sputter-coated with gold, and subsequently observed under an Apreo 2C field-emission scanning electron microscope (SEM; Thermo Scientific, Waltham, MA, USA) at 2 kV to evaluate the morphology and size of the microspheres. Additionally, 10 µL was transferred into a confocal dish, and fluorescence images were acquired using a laser scanning confocal microscope (LSCM; Nikon; ECLIPSE Ti2, Tokyo, Japan) with 488 nm laser excitation.

### 2.2. Animal

Male C57BL/6J mice, 21 days old and free from specific pathogens, were sourced from the Experimental Animal Center at Chongqing Medical University (license number SCXK [Yu] 2022-0010). The animals were maintained in individually ventilated cages under Specific Pathogen Free (SPF) conditions with free access to standard chow and water. Environmental conditions were strictly controlled: a 12 h light/dark cycle, relative humidity of 40–50%, and temperature maintained at 22 ± 2 °C. The Institutional Animal Care and Use Committee (IACUC) of the Children’s Hospital linked to Chongqing Medical University reviewed and approved all procedures in December 2024 (approval No. CHCMU-IACUC20241231007).

### 2.3. Experimental Protocol

The estimated daily intake of plastic particles for a 60 kg adult ranges from approximately 0.04 to 11.7 mg/kg/day [22,23,24]. Using body surface area normalization (human-to-mouse conversion factor Km ratio: 37/3), the mouse-equivalent dose was calculated as: mouse dose (mg/kg/day) = human dose (mg/kg/day) × (37/3). For a 0.02 kg mouse, this corresponds to ~0.01–2.88 mg/day [22]. Guided by this range, we selected administered doses of 10, 50, and 100 mg/kg/day, which translate to 0.2, 1.0, and 2.0 mg/day per 0.02 kg mouse, respectively. These doses fall within the calculated mouse-equivalent daily intake and represent low, medium, and high exposure levels. Male mice were randomly assigned to four groups (n = 10 per group): control, 10 mg/kg PS-NPs, 50 mg/kg PS-NPs, and 100 mg/kg PS-NPs. Prior to administration, polystyrene nanoplastics (PS-NPs) were dispersed in physiological saline by ultrasonication for 30 min and adjusted to the required concentrations. From postnatal day (PND) 22 to PND 35, mice received once-daily oral gavage, with the control group administered only physiological saline. On PND 35, animals were slaughtered, and testicular tissues were harvested for subsequent analyses.

### 2.4. Sample Collection

During the experimental period, body weight was recorded daily. Animals were maintained in a quiet, dry housing environment. Euthanasia was induced with carbon dioxide (CO_2_). The CO_2_ concentration was increased gradually in a sealed chamber until loss of consciousness. Terminal blood was collected by enucleation of the right eye, after which cervical dislocation was performed to ensure death. The weights of both testes were immediately measured.

Both testes were used to calculate the gonadosomatic index (GSI, %), defined as:

GSI (%) = [bilateral testis weight (g)/body weight (g)] × 100.

The left testis was immediately snap-frozen and stored at −80 °C. The right testis was subdivided for downstream analyses: one portion was immersed in 10% neutral buffered formalin (NBF) for histopathology, one portion was fixed in transmission electron microscopy (TEM) fixative for ultrastructural assessment, and one portion (n = 6 testicular tissue samples) was preserved in RNAlater for subsequent RNA sequencing. All animal procedures were reviewed and approved by the Experimental Animal Ethics Committee of Chongqing Medical University.

### 2.5. Hematoxylin and Eosin (H&E) Staining

The testicular tissues were preserved in 10% neutral buffered formalin for 24 h, then dehydrated using a series of graded ethanol, cleared with xylene, and embedded in paraffin. Poly-L-lysine-coated slides were used to mount sections that measured 4 μm in thickness. After deparaffinization and rehydration, slides were stained with hematoxylin and eosin (H&E), sealed, and examined under a Nikon optical microscope (Microscope; Nikon; ECLIPSE Ni-E, Tokyo, Japan) to evaluate morphological alterations.

### 2.6. TEM

Testicular tissues from the control, low-dose PS-NPs, mid-dose PS-NPs, and high-dose PS-NPs groups were fixed in 3% glutaraldehyde, dehydrated through a graded acetone series, sectioned into ultrathin slices (60–90 nm), and stained with uranyl acetate and lead citrate. Ultrastructural features were examined using a transmission electron microscope (TEM; JEOL JEM-1400Plus, Tokyo, Japan).

### 2.7. RNA Sequencing and Bioinformatic Analysis

Six testicular samples (n = 6)—three right testes from the control group and three right testes from the high-dose PS-NPs group—were submitted to LC-Bio (Hangzhou, China) for transcriptomic analysis. Messenger RNA libraries were prepared and sequenced on the Illumina NovaSeq 6000 platform. Differentially expressed genes (DEGs) were identified using edgeR and DESeq2 under consistent thresholds of |log_2_(fold change)| ≥ 1 and *p* < 0.05. Functional annotation and pathway enrichment were performed with Gene Ontology (GO), the Kyoto Encyclopedia of Genes and Genomes (KEGG), and Gene Set Enrichment Analysis (GSEA) [25,26].

### 2.8. Cell Culture and Treatment

The TM3 mouse Leydig cell line was obtained from Wuhan Puncell Life Technology Co., Ltd. (TM3; Procell system, CL-0234, Wuhan, China). The cells were cultured in Dulbecco’s modified Eagle medium/Ham’s F-12 (DMEM/F-12; Gibco, Waltham, MA, USA) with the addition of 10% fetal bovine serum (FBS; BDBIO, F801-050×10, Guangzhou, China) and 1% penicillin–streptomycin (NCM Biotech, C100C5, Shanghai, China) in a humidified incubator at 37 ± 0.5 °C with 5% CO_2_.

For exposures, cells were assigned to four groups: control and PS-NPs at 100, 200, or 400 μg/mL for 24 h. N-acetylcysteine (NAC; MCE, HY-B0215, Princeton, NJ, USA) and the NF-κB inhibitor 3-[(4-methylphenyl)sulfonyl]-2-propenenitrile (Bay 11-7082; MCE, HY-13453, Princeton, NJ, USA) were dissolved in dimethyl sulfoxide (DMSO) to prepare stock solutions. NAC was applied at 10 mM [27] and Bay 11-7082 at 5 μM [28], with co-treatments performed in combination with PS-NP suspensions for 24 h. The final DMSO concentration did not exceed 0.1% (*v*/*v*) in any treatment, and an equivalent vehicle control was included.

PTL (Parthenolide; MCE, HY-N0141, USA) was dissolved in DMSO to prepare a stock solution at a concentration of 10 μM [29]. The stock solution was then diluted with cell culture medium to achieve the desired working concentration. Subsequently, the existing cell culture medium was discarded prior to treatment, and the cells were exposed to the working solution of PTL for 24 h. The final concentration of DMSO in the treatment groups did not exceed 0.1% (*v*/*v*), and a solvent control group, with an equivalent volume of DMSO, was included in the experiment.

### 2.9. Ros

The levels of intracellular ROS in the cells were detected using the DCFH-DA probe (H2DCFDA, MCE, HY-D0940, USA), which is oxidized by ROS to produce green fluorescence. The fluorescence intensity is directly related to the intracellular ROS levels. First, the DCFH-DA probe was dissolved in DMSO to prepare a stock solution at a concentration of 10 mM. This stock solution was then diluted with medium at a ratio of 1:1000 to create a working solution. Subsequently, the culture medium in the dish was discarded, and the working solution was added to the cells, followed by incubation at 37 °C for 30 min. Finally, after washing three times with PBS, images were captured using a fluorescence microscope.

### 2.10. Western Blotting

Proteins from testicular tissue and cultured cells were extracted using radioimmunoprecipitation assay (RIPA) buffer (Beyotime, P0013B, Shanghai, China) supplemented with 1% phenylmethylsulfonyl fluoride (PMSF; Beyotime, ST506, China). Protein concentrations were determined by the bicinchoninic acid (BCA) assay (Beyotime, P0012, China). Equal amounts of protein were separated on 10% sodium dodecyl sulfate–polyacrylamide gels (SDS–PAGE) and transferred to polyvinylidene difluoride (PVDF) membranes using a wet-transfer system. Membranes were blocked with 5% bovine serum albumin (BSA) for 20 min and incubated with primary antibodies overnight at 4 °C with gentle rocking (antibody details in Table 1). After incubation with horseradish peroxidase (HRP)-conjugated secondary antibodies (1:20,000) at room temperature (RT) for 1 h, signals were developed using enhanced chemiluminescence (ECL; Millipore, WBULS0100, Burlington, MA, USA). Glyceraldehyde-3-phosphate dehydrogenase (GAPDH) served as the loading control. Band intensities were quantified by densitometry using Image Lab (v3.0, Bio-Rad, Hercules, CA, USA) and ImageJ (ImageJ 1.54g, National Institutes of Health, Bethesda, MD, USA). Table 1 details antibodies and their sources in a comprehensive manner.

### 2.11. Immunofluorescence (IF)

Glass coverslips in 24-well plates were used to seed TM3 cells. After treatments, cells were fixed using 4% paraformaldehyde (PFA) for 30 min, then blocked with 0.5% bovine serum albumin (BSA) at room temperature (RT) for an hour, and incubated with primary antibodies (1:100) overnight at 4 °C. Cells were then incubated with cyanine 3 (Cy3)-conjugated secondary antibodies (1:100) for an hour at RT, followed by nuclear counterstaining with Hoechst 33342 for 20 min. A Nikon fluorescence microscope (Nikon, Tokyo, Japan) was used to obtain fluorescence images.

For testicular tissue immunofluorescence, sections were deparaffinized in xylene, rehydrated through a graded ethanol series, subjected to microwave-assisted antigen retrieval in citrate buffer, and blocked with 0.5% BSA for 60 min at RT. Sections were incubated with primary antibodies (1:100) overnight at 4 °C, followed by Cy3-conjugated secondary antibodies (1:200) for 1 h at RT and Hoechst 33342 nuclear staining for 20 min. Images were captured using the same Nikon fluorescence microscope.

Testes were embedded in optimal cutting temperature (OCT) compound, rapidly frozen, cryosectioned at 10 μm, and mounted on slides. Nuclei were stained with Hoechst 33342 (Beyotime, C1022, China) for 25 min, and the localization of PS-NPs was examined by fluorescence microscopy (Nikon, Japan).

### 2.12. Reverse Transcription Quantitative PCR (RT–qPCR)

Total RNA was isolated from testicular tissue and TM3 cells using the MiniBEST Universal RNA Extraction Kit (TaKaRa, 9767, Tokyo, Japan). Complementary DNA (cDNA) was synthesized according to the manufacturer’s instructions with ABScript III RT Master Mix for qPCR (ABclonal, RK20429, Woburn, MA, USA). RT–qPCR was performed using 2× Universal SYBR Green Fast qPCR Mix (ABclonal, RK21203, USA) on a CFX96 real-time PCR detection system (Bio-Rad, USA). The 2^−ΔΔCt^ method was used to calculate relative mRNA levels, using glyceraldehyde-3-phosphate dehydrogenase (GAPDH) as the reference gene. The accession numbers are as follows: IL-1β gene: NM_008361.4; IL-6 gene: NM_031168.2; TNF-α gene: NM_013693.3; GAPDH gene: NM_008084.4. Primer sequences can be found in Table 2.

### 2.13. Determination of Testosterone, Malondialdehyde (MDA), and Glutathione (GSH) Levels

Blood was collected from each group, and serum testosterone was quantified by ELISA (Beyotime, PT872, China; limit of detection, LOD = 0.1 ng/mL; coefficient of variation, CV ≤ 5%) with absorbance read at 450 nm. For TM3 cells exposed to PS-NPs, supernatants of cell lysates were collected. MDA was measured using a commercial kit (Beyotime, S0131S, China) at 532 nm, and GSH was quantified with a glutathione assay kit (Elabscience, E-BC-K030-M, Wuhan, China) at 412 nm.

### 2.14. Co-Immunoprecipitation (Co-IP)

The interaction between p65 and steroidogenic factor-1 (SF-1) was examined using a Co-IP detection kit (Absin, abs955, Shanghai, China). TM3 cell lysates were incubated with anti-p65 antibody (1:100) at 4 °C overnight, followed by the addition of Protein A/G agarose beads to capture immune complexes. After spinning at 10,000× *g* for 5 min at 4 °C, the bead–antibody complexes were gathered and resuspended in 1× sodium dodecyl sulfate (SDS) loading buffer. Interacting proteins were subsequently analyzed by Western blotting, with SF-1 detected as the target protein. Normal IgG was used as a negative control to exclude nonspecific binding.

### 2.15. Drug Prediction and Molecular Docking

The Connectivity Map (CMap) is a comprehensive bioinformatics database that links small-molecule drugs, genes, and diseases through large-scale gene expression profiling [30,31]. Using CMap (https://clue.io/, 11 May 2025), potential prophylactic drugs for PS-NP-induced testicular injury were predicted by identifying small-molecule compounds with negative connectivity scores and evaluating their potential interaction sites with target proteins. This strategy enables the discovery of therapeutically promising compounds by comparing drug-induced gene expression signatures with those associated with testicular injury. Candidate compounds identified through CMap analysis were further evaluated by molecular docking and molecular dynamics simulations to predict their binding modes with target proteins. A high binding energy implies that the intermolecular interactions are weak and unstable. Moderate binding energy, ranging from −3 to −5 kcal/mol, suggests some level of stability but might require further optimization. A low binding energy, specifically below −5 kcal/mol, indicates strong and stable interactions, which are optimal for drug design as they forecast a tighter binding between a ligand and its target protein, suggesting increased therapeutic potential [32,33,34].

For molecular docking, AutoDock (AutoDock 4.2.6, Scripps Research Institute, La Jolla, CA, USA) software was used to calculate docking scores and assess binding affinities between small molecules and proteins. Molecular dynamics simulations were then performed to evaluate the stability of the compound–protein complexes, and binding free energies were estimated using the molecular mechanics/generalized Born surface area (MM/GBSA) method. Three-dimensional (3D) structural models of the drug–protein interactions were generated and visualized to illustrate the binding conformations.

### 2.16. Statistical Analysis

All experiments were independently repeated at least three times. Statistical analyses were performed using GraphPad Prism version 9.5 (GraphPad Software, San Diego, CA, USA). For transcriptomic analyses, multiple testing was controlled by the false discovery rate (FDR). Before planned parametric tests, data normality and homogeneity were evaluated using Shapiro–Wilk and Levene’s tests. Data are presented as mean ± standard deviation (SD). Two-group comparisons used unpaired *t* tests, and one-way analysis of variance (ANOVA) was applied for comparisons across multiple groups. When significant differences were detected, Tukey’s post hoc test was used for pairwise comparisons. A *p* value < 0.05 was considered statistically significant.

## 3. Results

### 3.1. Phenotypic Characteristics of PS-NPs

**Figure 1:**
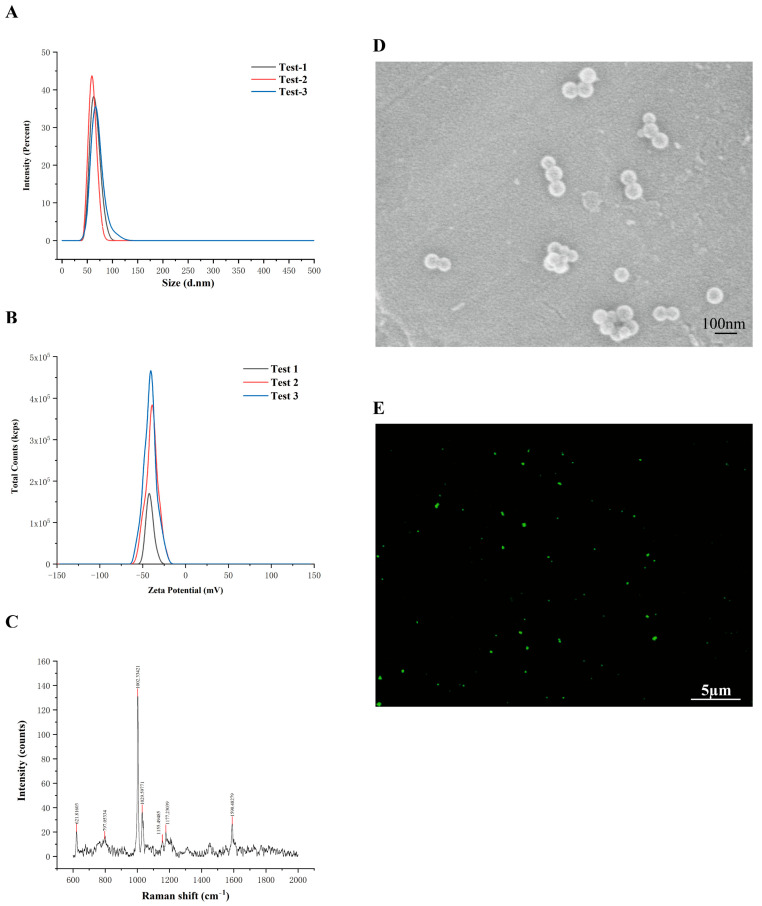
Characterization of PS-NPs. (**A**) Particle Size Analysis of PS-NPs (63.69 nm) (n = 3). (**B**) Zeta Potential of PS-NPs (−40.66 mV) (n = 3). (**C**) Raman Spectrum of PS-NPs. (**D**) SEM Image of PS-NPs. (**E**) Fluorescence Microscopy Image of PS-NPs.

DLS showed a Z-average diameter of 63.69 ± 3.21 nm, closely matching the nominal size (Figure 1A). The PDI was 0.25, indicating a relatively narrow size distribution. The mean ζ-potential was −40.66 ± 1.04 mV, consistent with high colloidal stability and a low tendency to aggregate (Figure 1B). After 1 month at room temperature, neither particle size nor PDI changed significantly, further supporting the formulation’s long-term stability. Raman spectroscopy confirmed the polystyrene composition of the particles, showing a characteristic peak at 1002.3 cm^−1^ (Figure 1C). SEM revealed that the PS-NPs were smooth, spherical, and uniformly distributed, with particle sizes consistent with the experimental specifications (Figure 1D). In addition, confocal microscopy demonstrated that green fluorescently labeled PS-NPs exhibited excellent dispersion within the culture medium (Figure 1E).

### 3.2. Exposure to PS-NPs Induces Structural Damage in Immature Mouse Testes

**Figure 2:**
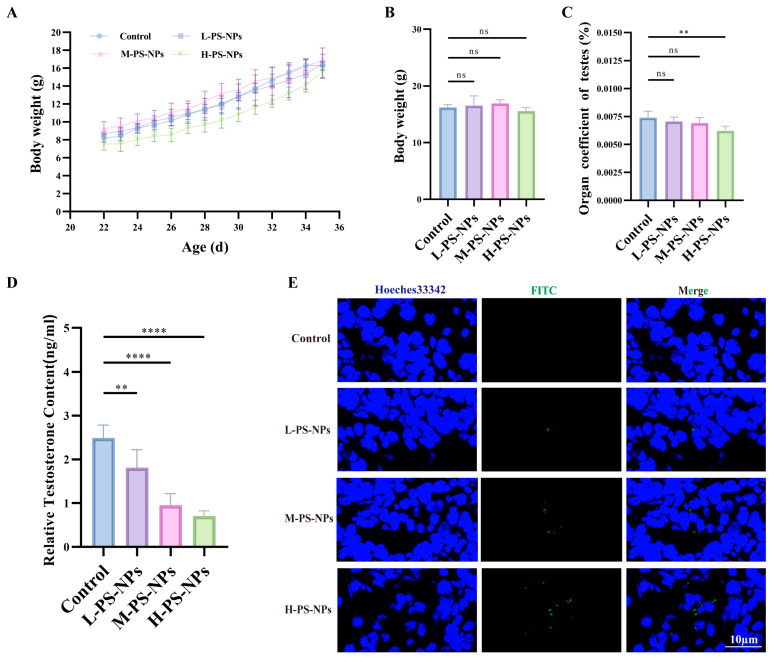
PS-NPs exposure on testicular health in immature mice. (**A**,**B**) Demonstrate no significant differences in body weight across the four groups (n = 10). (**C**) Indicates a reduction in the testis-to-body weight ratio in the high-dose group following PS-NPs exposure (n = 10). (**D**) Reveals a decline in serum testosterone levels, which is particularly pronounced in the high-dose group (n = 10). (**E**) Shows the accumulation of PS-NPs in testicular tissue via frozen sectioning. Statistical significance is denoted as ** *p* < 0.01, **** *p* < 0.0001.

A two-week oral exposure model was used to assess the impact of PS-NPs on immature mouse testes. No significant differences in body weight were detected between treatment and control groups (Figure 2A,B). In contrast, the testis-to-body weight ratio was significantly decreased in the high-dose group compared with controls (Figure 2C). Serum testosterone levels measured by ELISA showed a clear dose-dependent reduction, with the sharpest decline in the high-dose group (Figure 2D). No significant changes in luteinizing hormone (LH) or follicle-stimulating hormone (FSH) were detected throughout the study period (Figure S1). Fluorescent frozen sections revealed that green-labeled PS-NPs accumulated predominantly in interstitial cells in a concentration-dependent manner, whereas no signal was observed in controls (Figure 2E).

**Figure 3:**
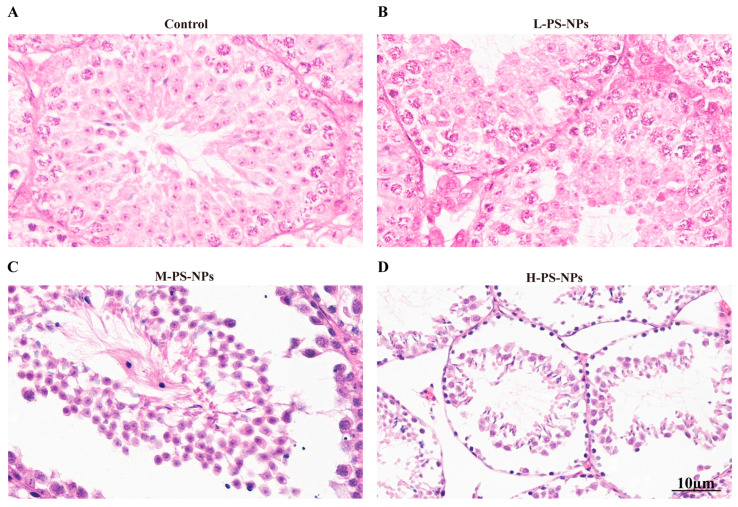
Effect of PS-NPs exposure on testicular pathology in immature mice. (**A**) The H&E staining of control testes displayed intact and well-organized seminiferous epithelia. (**B**) The low-dose group showed mild vacuolization in spermatogenic cells. (**C**) The medium-dose group exhibited pronounced vacuolization, seminiferous tubule atrophy, and interstitial expansion. (**D**) The high-dose group exhibited severe interstitial edema and pronounced seminiferous tubule atrophy.

H&E staining demonstrated progressive histopathological deterioration: control testes displayed intact, well-organized seminiferous epithelia (Figure 3A); the low-dose group showed mild vacuolization in spermatogenic cells (Figure 3B); the medium-dose group exhibited pronounced vacuolization, seminiferous tubule atrophy, and interstitial expansion (Figure 3C); and the high-dose group presented with severe interstitial edema accompanied by marked seminiferous tubule atrophy (Figure 3D).

**Figure 4:**
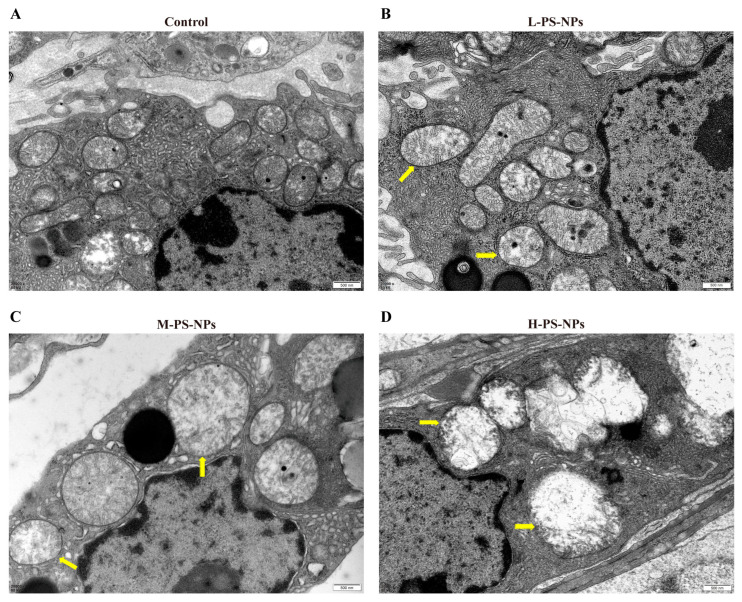
Effect of PS-NPs exposure on testicular ultrastructure in immature mice. (**A**) Provides TEM ultrastructural evidence that controls exhibited intact mitochondria and preserved organelles. (**B**) In the low-dose group, mitochondria showed partial matrix clearing and mild cristae swelling. (**C**) The medium-dose group exhibited a cloudy matrix with fragmented and shortened cristae. (**D**) In the high-dose group, the matrix was fully cleared and cristae were disintegrated, with yellow arrows indicating damaged mitochondria.

TEM analysis confirmed ultrastructural disruption in Leydig cells. Controls showed intact mitochondria and preserved organelles (Figure 4A), while PS-NP exposure caused progressively severe mitochondrial damage. In the low-dose group, mitochondria exhibited partial matrix clearing and mild cristae swelling (Figure 4B). In the medium-dose group, the matrix appeared cloudy and cristae were fragmented and shortened (Figure 4C). In the high-dose group, the matrix was completely cleared and cristae were disintegrated (Figure 4D).

Together, these results demonstrate that PS-NP exposure induces dose-dependent testicular damage in immature mice, reflected in both histopathological alterations and functional impairment.

### 3.3. Transcriptomic Analysis Reveals PS-NPs-Induced Impairment of Testicular Testosterone Synthesis

**Figure 5:**
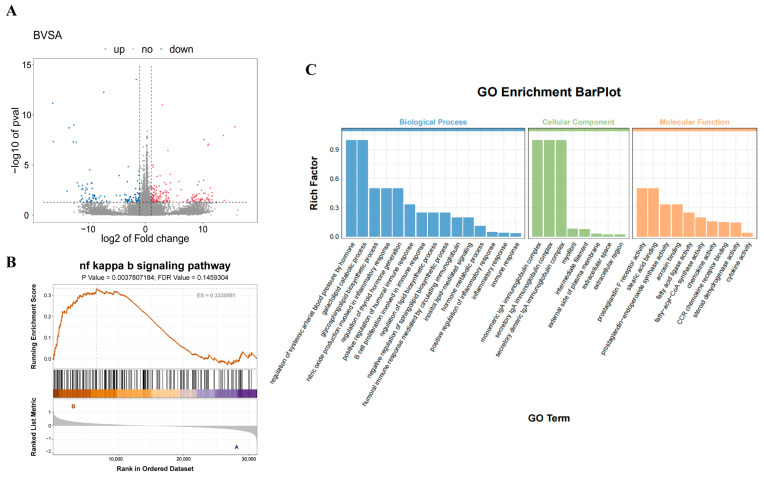
Transcriptomic analysis of control versus PS-NPs groups. (**A**) The volcano plot illustrates the DEGs between the control and PS-NPs groups, 174 upregulated and 112 downregulated genes (n = 3). (**B**) GO functional annotation enrichment analysis of DEGs. (**C**) GSEA revealed significant enrichment of the NF-κB signaling pathway among the DEGs.

To clarify the molecular mechanisms of PS-NPs-induced testicular injury, transcriptomic profiling was performed on mouse testes. Comparative analysis between the control and PS-NP groups identified 286 DEGs, including 174 upregulated and 112 downregulated transcripts (Figure 5A). GO annotation revealed enrichment in biological processes such as lipid biosynthetic regulation, positive regulation of inflammatory response, and inflammatory response, alongside molecular functions including fatty acid ligase activity, fatty acyl-CoA synthase activity, and steroid dehydrogenase activity, suggesting disruption of lipid metabolism in immature testes (Figure 5C). GSEA further demonstrated significant enrichment of the NF-κB signaling pathway (Figure 5B), implicating its involvement in testicular injury.

**Figure 6:**
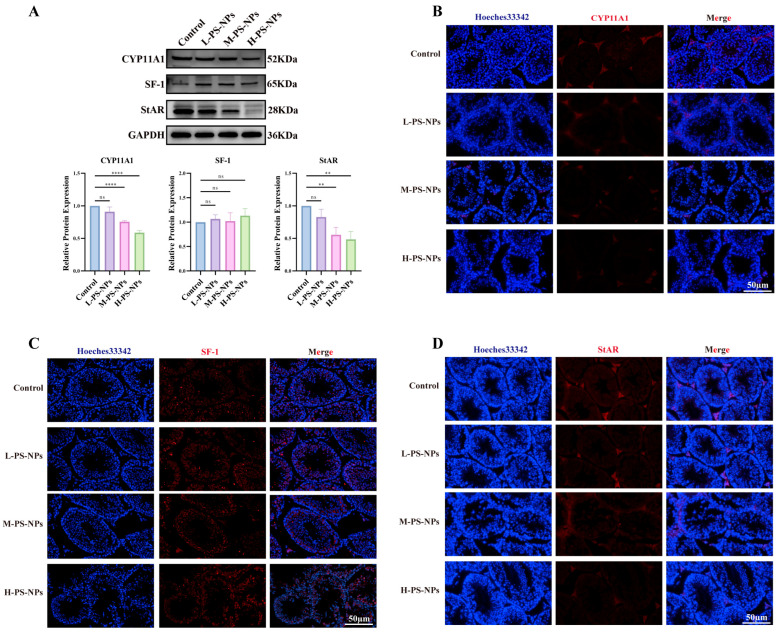
Testicular testosterone synthesis is impaired in the PS-NPS group. (**A**) Proteins involved in testosterone synthesis, specifically CYP11A1 and StAR, were downregulated in the PS-NPs groups compared to controls, whereas SF-1 expression remained unchanged (n = 3). (**B**) Immunofluorescence analysis demonstrated a reduction in CYP11A1 expression in the PS-NPs groups. (**C**) Immunofluorescence analysis indicated no significant change in SF-1 expression. (**D**) Immunofluorescence analysis showed decreased levels of StAR in the PS-NPs groups. Statistical significance is denoted as ** *p* < 0.01, **** *p* < 0.0001.

Western blotting was then applied to assess proteins involved in testosterone biosynthesis. CYP11A1 and StAR showed dose-dependent decreases with increasing PS-NP exposure, whereas SF-1 expression remained unchanged (Figure 6A). Immunofluorescence assays confirmed these findings, showing reduced CYP11A1 and StAR staining intensity in exposed testes (Figure 6B–D). Collectively, these results indicate that PS-NP exposure suppresses testosterone synthesis and contributes to germ cell injury in immature mice.

### 3.4. PS-NPs Activate the ROS/NF-κB Pathway in TM3 Cells and Reduce Testosterone Production

**Figure 7:**
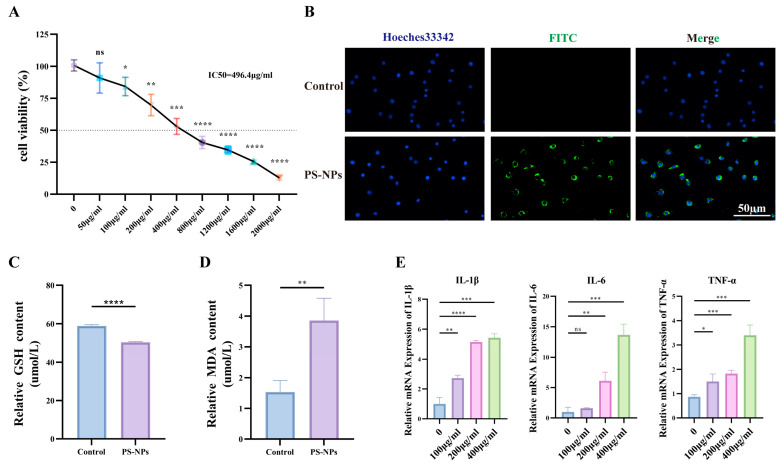
PS-NPs exposure can damage the TM3 cell line. (**A**) Exposure to varying concentrations of PS-NPs for 24 h resulted in a concentration-dependent inhibition of TM3 cell proliferation. (**B**) Morphological assessment of TM3 cell lines following PS-NPs treatment. (**C**) Alterations in GSH levels post PS-NPs exposure (n = 3). (**D**) Modifications in MDA levels subsequent to PS-NPs treatment (n = 3). (**E**) Expression analysis of mRNA for inflammation-related genes in TM3 cell lines (n = 3). Statistical significance is denoted as * *p* < 0.05, ** *p* < 0.01, *** *p* < 0.001, **** *p* < 0.0001.

TM3 cells were used as an in vitro Leydig model. PS-NPs suppressed cell proliferation in a concentration-dependent manner (IC50 = 496.4 μg/mL); at 100 μg/mL, viability remained >80% and was selected as the working dose (Figure 7A). Uptake of green-labeled PS-NPs was evident after exposure to 100 μg/mL (Figure 7B). Exposure to PS-NPs at 100 μg/mL significantly increased the cellular fluorescence intensity compared to the control group, which indicates elevated levels of ROS (Figure S2). Relative to controls, PS-NPs (100 μg/mL) depleted GSH (Figure 7C) and increased MDA (Figure 7D), consistent with ROS induction. To examine dose dependency, cells were treated with 0, 100, 200, or 400 μg/mL PS-NPs for 24 h. We analyzed the expression levels of inflammation-related genes. The results indicated that the expression levels of IL-6, IL-1β, and TNF-α significantly increased with increasing concentrations of PS-NPs. This demonstrated a dose-dependent response (Figure 7E).

**Figure 8:**
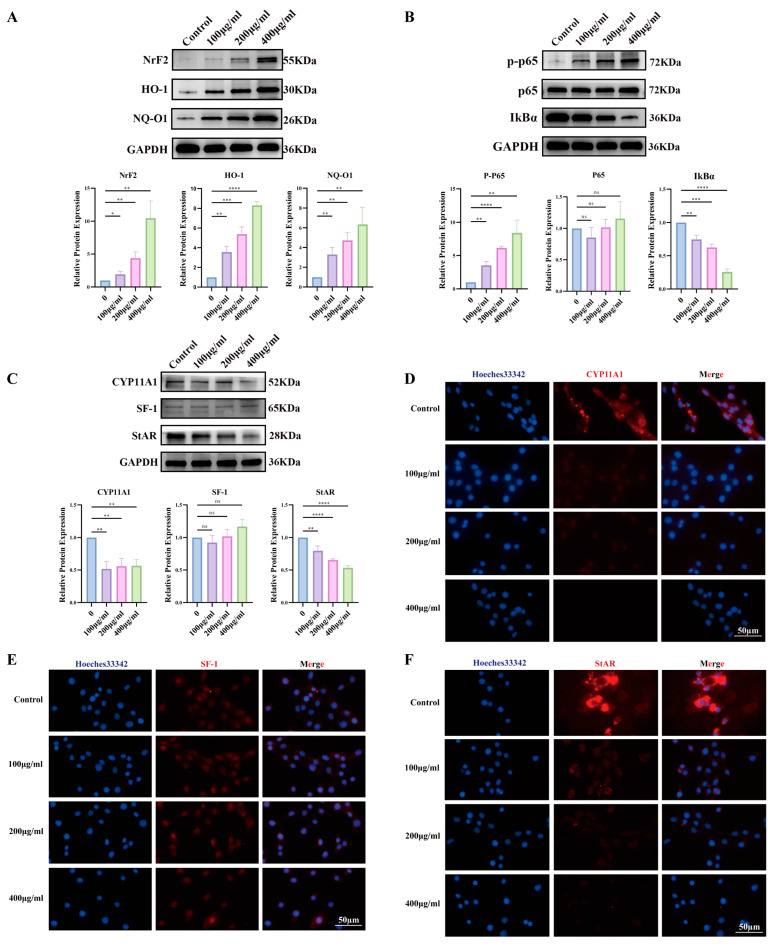
Activation of the ROS/NF-κB signaling pathway by PS-NPs exposure in TM3 cell lines results in decreased testosterone production. (**A**) Variations in oxidative stress-related protein levels following treatment with different concentrations of PS-NPs (n = 3). (**B**) Alterations in NF-κB signaling pathway-related protein levels after exposure to varying concentrations of PS-NPs (n = 3). (**C**) Modifications in proteins associated with testosterone production following treatment with different concentrations of PS-NPs (n = 3). (**D**–**F**) Immunofluorescence analysis of testosterone production-related proteins CYP11A1, SF-1, and StAR in TM3 cells. Statistical significance is denoted as * *p* < 0.05, ** *p* < 0.01, *** *p* < 0.001, **** *p* < 0.0001.

To examine dose dependency, cells were treated with 0, 100, 200, or 400 μg/mL PS-NPs for 24 h, accompanied by upregulation of oxidative-stress proteins (Figure 8A). GSEA based on DEGs indicated activation of the NF-κB pathway (Figure 8B).

Steroidogenic markers showed dose-dependent decreases in CYP11A1 and StAR, whereas SF-1 remained unchanged (Figure 8C). IF corroborated these results, showing reduced CYP11A1 (Figure 8D) and StAR (Figure 8E) signals across doses, while SF-1 was stable (Figure 8F). Collectively, PS-NPs activate ROS/NF-κB signaling in TM3 cells and blunt testosterone biosynthesis.

### 3.5. NAC Mitigates PS-NP-Induced ROS/NF-κB Activation and Restores Testosterone Synthesis

**Figure 9:**
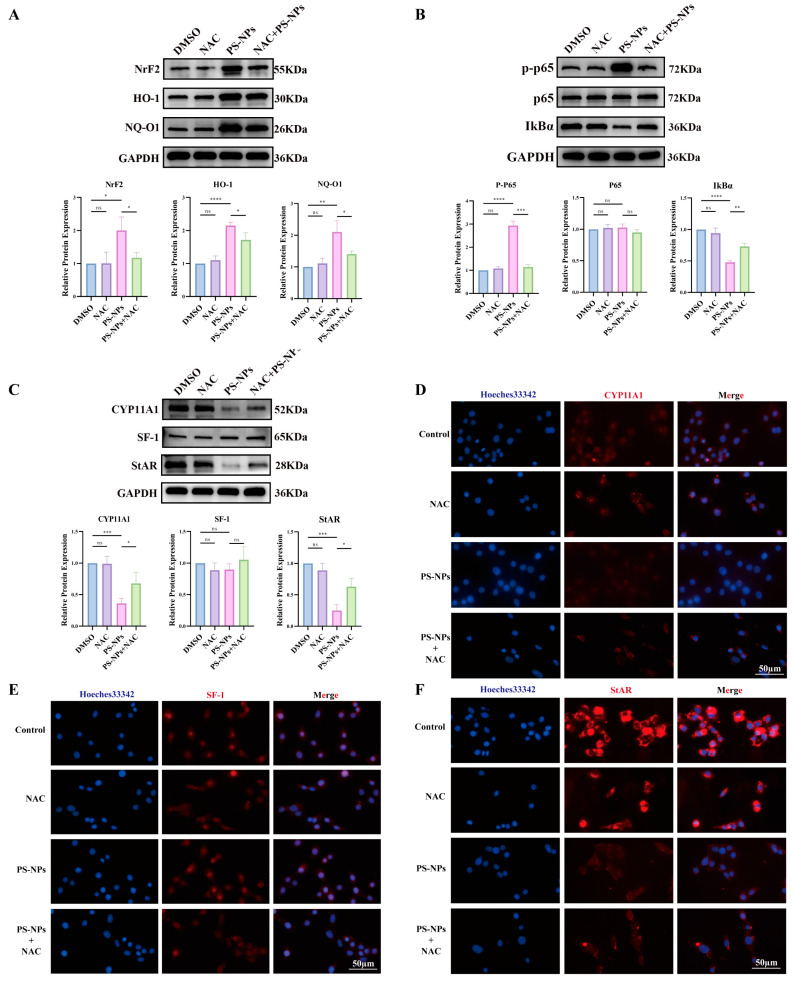
NAC is an inhibitor of the ROS/NF-κB signaling pathway and can restore testosterone production. (**A**) NAC effectively inhibits the expression of oxidative stress-related proteins (n = 3). (**B**) NAC suppresses the NF-κB signaling pathway (n = 3). (**C**) NAC restores the production of proteins associated with testosterone synthesis (n = 3). (**D**–**F**) Immunofluorescence analyses of testosterone production-related proteins, which are rescued by the NAC inhibitor. Statistical significance is denoted as * *p* < 0.05, ** *p* < 0.01, *** *p* < 0.001, **** *p* < 0.0001.

To test whether quenching ROS rescues steroidogenesis, NAC (10 mM) was applied during PS-NPs exposure. NAC reduced oxidative-stress protein levels (Figure 9A), suppressed NF-κB activation (Figure 9B), and upregulated steroidogenic proteins (Figure 9C). IF corroborated these results, showing increased CYP11A1, SF-1, and StAR signals in NAC-treated cells compared with PS-NPs alone (Figure 9D–F). Collectively, NAC attenuates PS-NP-driven ROS/NF-κB signaling and partially restores testosterone biosynthesis.

### 3.6. Bay 11-7082 Inhibits NF-κB Activation and Restores Testosterone Synthesis

**Figure 10:**
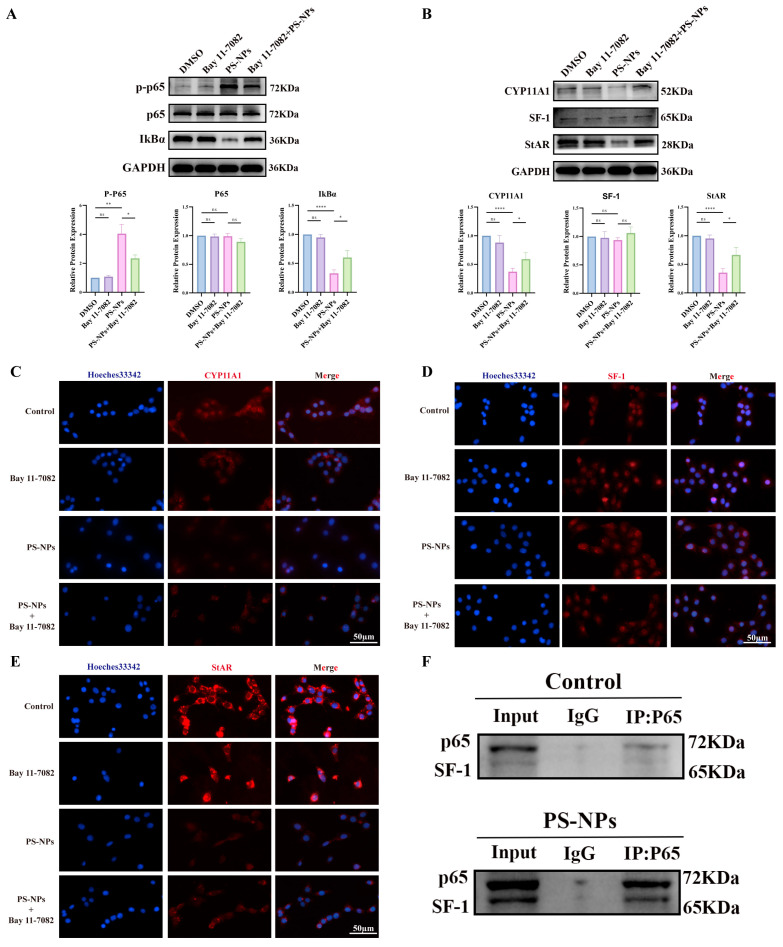
Bay 11-7082 is an inhibitor of the NF-κB signaling pathway and can restore testosterone production. (**A**) Bay 11-7082 inhibits the NF-κB signaling pathway (n = 3). (**B**) Bay 11-7082 restores the production of testosterone-related proteins (n = 3). (**C**–**E**) Immunofluorescence analyses of testosterone production-related proteins, which are rescued by the Bay 11-7082 inhibitor. (**F**) Co-IP analysis of the interaction between p65 and SF-1 in TM3 cells (n = 3). Statistical significance is denoted as * *p* < 0.05, ** *p* < 0.01, **** *p* < 0.0001.

To further test the role of NF-κB in steroidogenesis, Bay 11-7082 (5 μM) was applied during PS-NPs exposure. Bay 11-7082 suppressed NF-κB activation (Figure 10A) and increased steroidogenic protein expression (Figure 10B). IF confirmed higher levels of CYP11A1, SF-1, and StAR in Bay 11-7082-treated cells compared with PS-NPs alone (Figure 10C–E). These results indicate that Bay 11-7082 mitigates PS-NP-induced NF-κB signaling and restores testosterone production.

To probe the endogenous link between NF-κB and steroidogenesis, Co-IP assays were performed for p65 and SF-1 (Figure 10F). The data showed that NF-κB activation strengthened p65–SF-1 binding, which was associated with reduced testosterone synthesis.

### 3.7. PS-NPs Activate the ROS/NF-κB Pathway in Testicular Tissue

**Figure 11:**
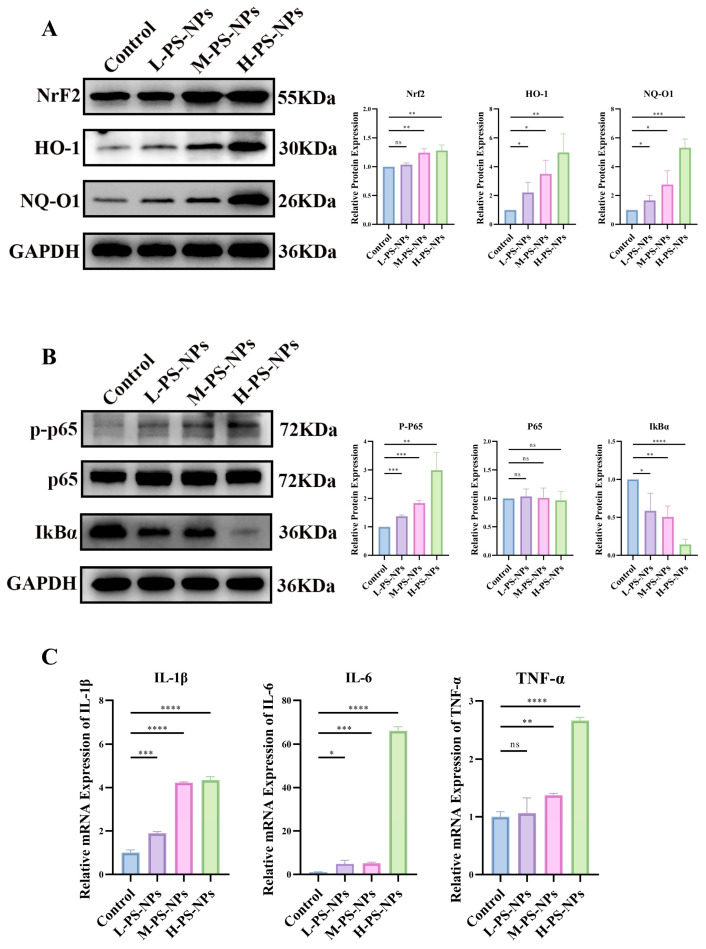
Exposure to PS-NPs Activates the ROS/NF-κB Signaling Pathway in Testicular Tissue. (**A**) ROS levels in testicular tissue exhibited a dose-dependent significant increase following PS-NPs exposure (n = 3). (**B**) The NF-κB signaling pathway was activated in the testicular tissue (n = 3). (**C**) mRNA expression levels of inflammation-related genes in testicular tissue were assessed (n = 3). Statistical significance is denoted as * *p* < 0.05, ** *p* < 0.01, *** *p* < 0.001, **** *p* < 0.0001.

Consistent with prior reports [35]. We assessed ROS-related signaling in testes after PS-NP exposure. Relative to controls, increasing PS-NP doses elicited stepwise upregulation of NRF2, HO-1, and NQO1 in testicular tissue, indicating a dose-dependent ROS response (Figure 11A). Guided by the transcriptomic findings, we next examined NF-κB activity. PS-NPs increased p-p65 and decreased IκBα compared with controls, confirming NF-κB activation in testes (Figure 11B). In parallel, transcripts of pro-inflammatory genes were elevated in germ cells following PS-NPs exposure (Figure 11C). Together, these data demonstrate that PS-NPs trigger a ROS surge and activate NF-κB signaling in immature testicular tissue.

### 3.8. PTL Rescues PS-NP-Induced Testicular Damage

**Figure 12:**
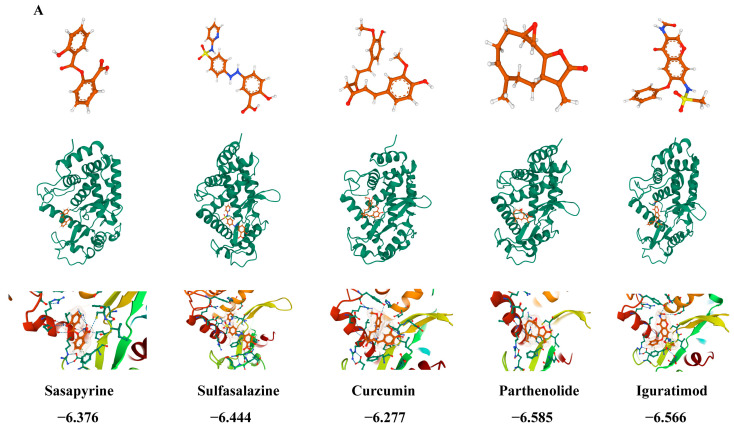

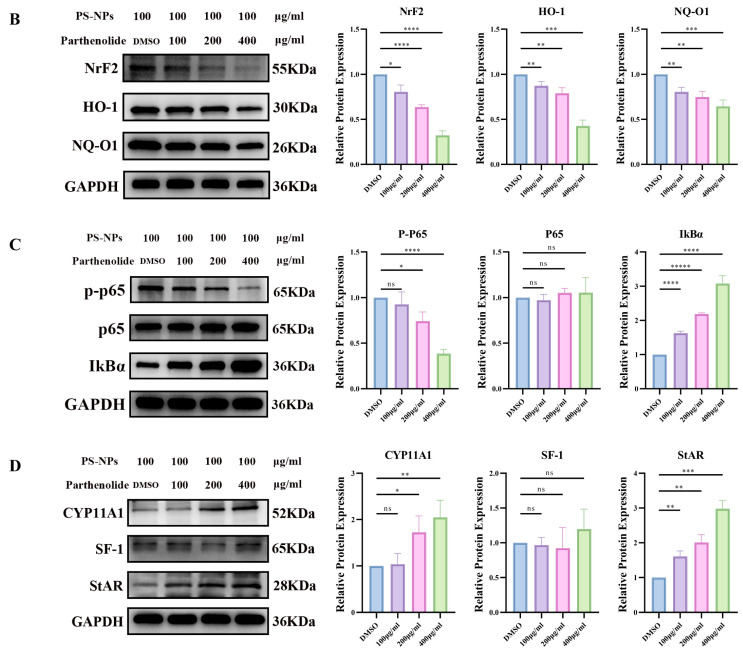
Drug screening utilizing testicular transcriptome sequencing. (**A**) Five small-molecule compounds were identified through cMAP screening, with PTL demonstrating the highest binding affinity (−6.585 kcal/mol). (**B**) PTL was found to inhibit proteins associated with oxidative stress (n = 3). (**C**) PTL also suppressed proteins related to the NF-κB signaling pathway (n = 3). (**D**) Furthermore, PTL restored proteins involved in testosterone production (n = 3). Statistical significance was noted as * *p* < 0.05, ** *p* < 0.01, *** *p* < 0.001, **** *p* < 0.0001, ***** *p* < 0.00001.

Upregulated DEGs in testes (n = 174) were submitted to CMap. Compounds with negative connectivity included salsalate, sulfasalazine, curcumin, PTL, and iguratimod, suggesting potential to reverse the injury signature. Docking prioritized PTL, which showed the strongest predicted binding among candidates (Figure 12A). CCK-8 showed no overt PTL cytotoxicity at the tested doses: cell viability remained >90%, indicating that the exposure conditions were well tolerated and did not induce significant toxicity (Figure S3).

In TM3 cells pre-exposed to PS-NPs (100 μg/mL), PTL given over graded doses reduced oxidative-stress markers (WB; Figure 12B), suppressed NF-κB activation (WB; Figure 12C), and restored testosterone output (WB; Figure 12D). Collectively, these data support PTL as a candidate to counter PS-NP-induced reproductive injury.

## 4. Discussion

NPs (<1 μm) have emerged as pervasive contaminants with documented biological effects. In this study, 60 nm green-fluorescent PS-NPs were administered by oral gavage to immature mice. Integrated transcriptomic and experimental evidence showed that PS-NPs provoke synergistic toxicity in prepubertal testes, characterized by disrupted lipid metabolism in LCs and activation of the ROS/NF-κB axis. Moreover, PTL was identified as a candidate small molecule that partially restores testosterone secretion in PS-NP-exposed TM3 cells.

PS-NPs can cross biological barriers, accumulate in tissues, and elicit multi-organ toxicity. Reported outcomes include GI injury with microbial dysbiosis and heightened infection susceptibility, hepatic and renal oxidative damage [36,37,38], and potential cardiovascular perturbations suggested by omics profiling [39]. Neurotoxicity—particularly during development—has also been observed, with neuronal injury and behavioral abnormalities [40]. Within the reproductive system, PS-NPs impair germ cells and endocrine function: in males, they reduce sperm quality and perturb reproductive hormones through ROS and inflammation [41]; in female fish, they downregulate steroidogenic genes and compromise fecundity [42]. Consistent with these data, our results show that PS-NPs depress testosterone secretion and induce testicular injury in prepubertal mice. These observations suggest that reduced testosterone is unlikely to stem from HPG-axis dysfunction and is more directly attributable to intratesticular defects in steroidogenesis. The findings underscore a central role for ROS in nanoplastic-induced reproductive effects and warrant deeper investigation of intratesticular mechanisms, including the specific contribution of NF-κB to testosterone biosynthesis.

Oxidative stress is a central driver of cellular injury across species. In zebrafish embryos, NPs trigger ROS accumulation and elevations of SOD and CAT, contributing to developmental toxicity [43]. Prior work indicates that PS-NPs impair reproduction by increasing ROS [44], although mechanisms remain incompletely defined. Here, we observed robust ROS-related responses in vivo and in vitro, and NAC restored NF-κB signaling and testosterone output, supporting oxidative stress as a primary mediator of PS-NP-induced testicular injury.

NF-κB orchestrates immune and inflammatory programs and is activated by ROS via multiple routes [11,45,46]. First, ROS can directly stimulate NF-κB; cross-talk with Nrf2 modulates inflammation and redox injury [47]. In metabolic disease, oxidative stress drives inflammation and apoptosis through NF-κB [48]. Second, ROS indirectly activate NF-κB by engaging upstream cascades; natural compounds (e.g., Akebia saponin D, isoliquiritigenin) alleviate ROS-driven fibrosis by suppressing NF-κB [48,49]. Third, ROS-responsive pathways including MAPK and PI3K/Akt converge on NF-κB [50,51]. Disease models further substantiate this axis: renal I/R engages TLR4/TRAF6/NF-κB to promote inflammation [52], while PI3K/Akt/GSK-3β/NF-κB mediates lung injury [53]. In line with these paradigms, our data indicate that PS-NPs activate NF-κB via ROS.

NF-κB also influences steroid hormone biosynthesis. In females, nanoparticle exposure alters ovarian CYP450 expression via NF-κB, perturbing hormone metabolism [54]. In males, obesity increases testicular ROS and inflammation, suppressing steroidogenic enzymes through NF-κB and lowering serum testosterone with compromised sperm quality [55]; in diabetes, NF-κB activation reduces transcripts required for testosterone synthesis, while anti-inflammatory/antioxidant interventions reverse these effects [56]. Metals such as cadmium engage TLR4/MAPK/NF-κB to damage the testis and blunt testosterone output [57]. MPs inhibit LHR transcription through NF-κB, reducing testosterone synthesis and secretion [58]. Mechanistically, p65 can bind SF-1 and recruit HDACs, repressing SF-1-dependent transcription [59]. Concordantly, we observe enhanced p65–SF-1 interaction with PS-NPs exposure, accompanied by reduced CYP11A1 and StAR, linking NF-κB activity to impaired steroidogenesis.

NF-κB activity is further intertwined with cholesterol metabolism—the precursor supply for steroidogenesis. LPS-driven neuroinflammation modulates cholesterol turnover via TLR4/MyD88/NF-κB, accelerating catabolism and suppressing synthesis (e.g., CYP46A1, HMGCR) [60]. Proteomics from BYHW-treated ischemic heart failure implicates cholesterol metabolism and NF-κB signaling [61]. Disturbed cholesterol handling can trigger NF-κB-dependent damage; for example, SR-BI deficiency promotes iron overload and ferroptosis via PKC-β/NF-κB [62]. Together with our findings, these observations support a model in which PS-NPs activate ROS/NF-κB, p65 associates with SF-1, and steroidogenic enzymes decline, culminating in reduced testosterone secretion.

CMap screening prioritized PTL, predicting the strongest binding among candidates and nominating it as a ROS/NF-κB-targeting compound. PTL is widely recognized for anti-inflammatory, antioxidant, and anti-apoptotic actions via NF-κB suppression [63,64]. Our data indicate that PTL increases testosterone in LCs by dampening ROS and inhibiting NF-κB in a concentration-dependent manner, supporting its potential as a prophylactic agent against PS-NP-induced reproductive toxicity.

This study has limitations. First, we modeled acute exposure at the upper bound of environmental concentrations in immature mice; outcomes may differ from chronic human exposure. Future work will employ low-dose, long-term paradigms. Second, mechanistic validation was largely performed in cells. Although PTL rescued LC injury in vitro, in vivo efficacy, biodistribution, and safety remain to be determined. Third, beyond LCs, potential impacts on GCs and SCs warrant systematic evaluation. Addressing these gaps will strengthen translational relevance. Finally, this study did not perform p65 knockdown to validate the specific role of the NF-κB pathway in ROS-induced suppression of testosterone. Although we provided preliminary evidence by measuring ROS and NF-κB activation, the absence of direct gene knockdown limits the conclusiveness of this mechanism. Future work should employ p65 knockdown (e.g., siRNA/CRISPR) or complementary molecular approaches to delineate NF-κB’s contribution to steroidogenesis.

## 5. Conclusions

This study demonstrates that PS-NPs cause testicular injury in both acute mouse models and LC models, with NF-κB signaling acting as a key mediator. We provide the first evidence directly linking ROS/NF-κB activation to PS-NP-induced damage in immature testes. Enhanced p65–SF-1 interaction emerged as a mechanistic axis connecting NF-κB signaling to impaired T biosynthesis. Furthermore, PTL was identified as a candidate compound that alleviates PS-NP-induced injury, offering new perspectives for preventing and treating nanoplastic-driven reproductive toxicity.

## Figures and Tables

**Table 1:** Antibodies and their sources.

Antibodies Application	Application	Campany
CYP11A1	WB (1:1000), IF (1:100)	13363-1-AP, Proteintech (Rosemont, IL, USA)
SF-1	WB (1:500), IF (1:100)	18658-1-AP, Proteintech (Rosemont, IL, USA)
StAR	WB (1:500), IF (1:100)	8449T, Cell Signaling (Danvers, MA, USA)
NRF2	WB (1:1000), IF (1:100)	16396-1-AP, Proteintech (Rosemont, IL, USA)
HO-1	WB (1:1000), IF (1:100)	10701-1-AP, Proteintech (Rosemont, IL, USA)
NQ-O1	WB (1:1000), IF (1:100)	11451-1-AP, Proteintech (Rosemont, IL, USA)
P-P65	WB (1:1000), IF (1:100)	3033T, Cell Signaling (Danvers, MA, USA)
P65	WB (1:1000), IF (1:100)	10745-1-AP, Proteintech (Rosemont, IL, USA)
IkBα	WB (1:1000), IF (1:100)	10268-1-AP, Proteintech (Rosemont, IL, USA)
GAPDH	WB (1:20,000)	10494-1-AP, Proteintech (Rosemont, IL, USA)
goat anti-mouse	WB (1:20,000)	RGAM001, Proteintech (Rosemont, IL, USA)
goat anti-rabbit	WB (1:20,000)	RGAG001, Proteintech (Rosemont, IL, USA)
Cy3-conjugated goat anti-mouse antibody	IF (1:200)	SA00009-1, Proteintech (Rosemont, IL, USA)
Cy3-conjugated goat anti-rabbit antibody	IF (1:200)	SA00009-2, Proteintech (Rosemont, IL, USA)
FITC-conjugated mouse anti-mouse antibody	IF (1:200)	SA00003-1, Proteintech (Rosemont, IL, USA)

**Table 2:** Primers used for real-time PCR.

Gene	Primers	Sequence 5′→3′
IL-1β	Sense	TGCAAGTGTCTGAAGCAGCTATG
	Antisense	TAGCCACTGAAGGAGATGAGTTG
IL-6	Sense	AACCGCTATGAAGTTCCTCTCTG
	Antisense	TGGTATCCTCTGTGAAGTCTCCT
TNF-α	Sense	CCAGACCCTCACACTCAGATCAT
	Antisense	AGAACCTGGGAGTAGACAAGGTA
GAPDH	Sense	AGGTCGGTGTGAACGGATTTG
	Antisense	TGTAGACCATGTAGTTGAGGTCA

## Data Availability

The original contributions presented in this study are included in the article/Supplementary Materials. Further inquiries can be directed to the corresponding author.

## Supplementary Materials

The following supporting information can be downloaded at: https://www.mdpi.com/article/10.3390/antiox14111315/s1, Figure S1. Alterations in serum LH and FSH levels in immature mice following exposure to PS-NPs. (A) The serum LH levels showed no significant differences between the groups (n = 10). (B) The serum FSH levels showed no significant differences between the groups (n = 10). Statistical significance is denoted as * *p* < 0.05, ** *p* < 0.01, *** *p* < 0.001. Figure S2. ROS detection in TM3 cells. Exposure to PS-NPs at 100 μg/mL significantly increased the cellular fluorescence in-tensity compared to the control group, which indicates elevated levels of ROS. Figure S3. The CCK-8 assay was used to evaluate the cytotoxicity of PTL. Within the experimental dose range, PTL treatment did not significantly affect cell viability, which remained above 90%. Statistical significance is denoted as * *p* < 0.05, ** *p* < 0.01, *** *p* < 0.001.
