# Supplementary material for: Parthenolide Restores Testosterone Biosynthesis After Nanoplastic Exposure by Blocking ROS-Driven NF-κB Nuclear Translocation
Source: Antioxidants (Basel). 2025 Oct 31;14(11):1315. doi: 10.3390/antiox14111315 (PMC12649254; doi:10.3390/antiox14111315)
Supplement: Supplementary file 1 [file antioxidants-14-01315-s001.zip › antioxidants-3916828-supplementary.pdf]

Supplementary information

for

Parthenolide Restores Testosterone Biosynthesis after Nanoplastic Exposure by Blocking ROS-Driven NF-κB Nuclear Translocation

Peng Zhao[1,2], Hao Yan[1,2], Runchang Wang[1,2], Jie Zhao[1,2], Xiangqin Zheng[1,2], Dinggang Li[1,2], Xitong Guo[1,2], Fengming Ji[1,2],Chunlan Long[1,2], Lianju Shen[1,2], Guanghui Wei[1,2], Shengde Wu[1,2,*]

[1.] **Department of Urology, Children's Hospital of Chongqing Medical University, National Clinical Research Center for Child Health and Disorders, Ministry of Education Key Laboratory of Child Development and Disorders, Chongqing, 400014, PR China.**

[2.] **Children Urogenital Development and Tissue Engineering of Chongqing Education Commission of China, Chongqing, 400014, PR China.**

*** Correspondence: shengdewu@hospital.cqmu.edu.cn**

Content:

1. Alterations in serum LH and FSH levels in immature mice following exposure to PS-NPs. pg 10; line 350-352

2. ROS detection in TM3 cells. pg 14; line 427-429

3. The CCK-8 assay was used to evaluate the cytotoxicity of PTL. pg 20; line 513-516

1. Alterations in serum LH and FSH levels in immature mice following exposure to PS-NPs.

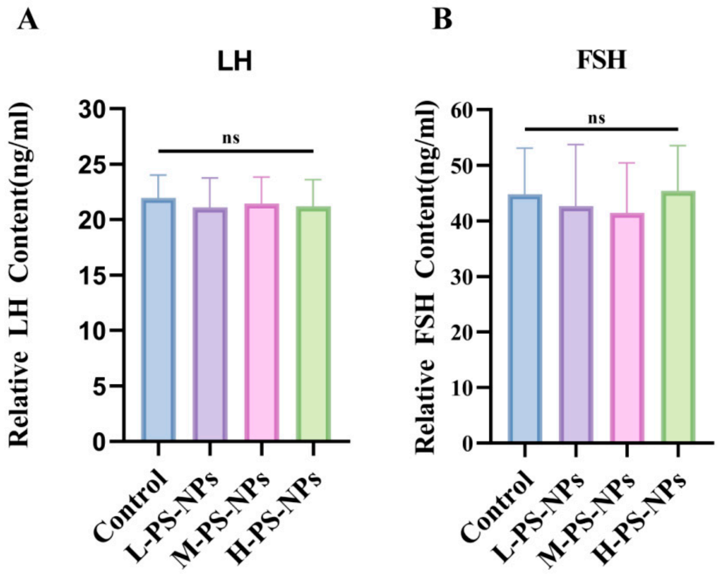

Figure S1. Alterations in serum LH and FSH levels in immature mice following exposure to PS-NPs. (A) The serum LH levels showed no significant differences between the groups (n=10). (B) The serum FSH levels showed no significant differences between the groups (n=10). Statistical significance is denoted as *$p < 0.05$, **$p < 0.01$, ***$p < 0.001$.

2. ROS detection in TM3 cells.

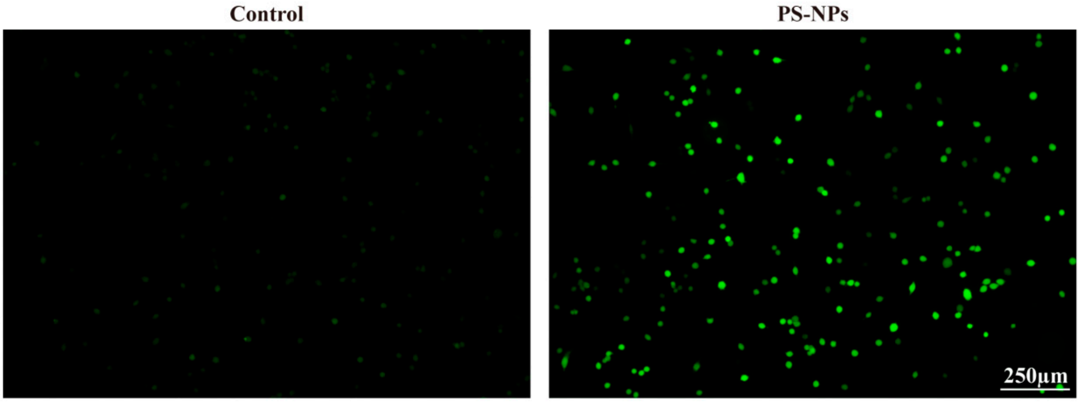

Figure S2. ROS detection in TM3 cells. Exposure to PS-NPs at 100 μg/mL significantly increased the cellular fluorescence in-tensity compared to the control group, which indicates elevated levels of ROS.

3. The CCK-8 assay was used to evaluate the cytotoxicity of PTL.

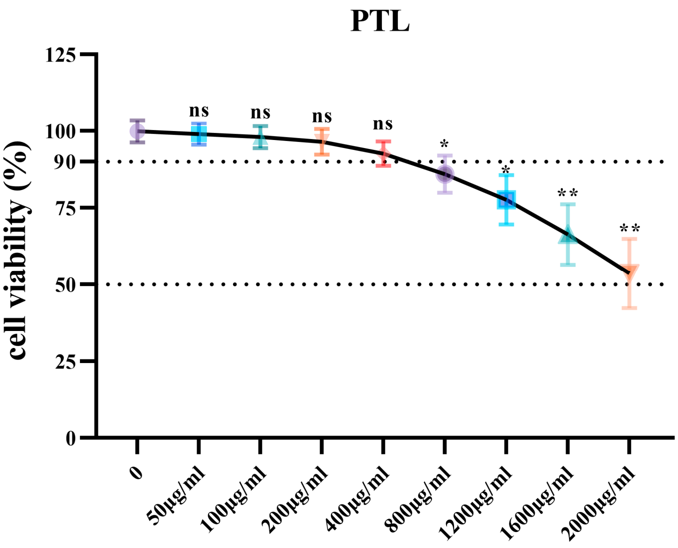

Figure S3. The CCK-8 assay was used to evaluate the cytotoxicity of PTL. Within the experimental dose range, PTL treatment did not significantly affect cell viability, which remained above 90%. Statistical significance is denoted as *$p < 0.05$, **$p < 0.01$, ***$p < 0.001$.
